# Radiation-induced effects and the immune system in cancer

**DOI:** 10.3389/fonc.2012.00191

**Published:** 2012-12-17

**Authors:** Punit Kaur, Alexzander Asea

**Affiliations:** Department of Microbiology, Biochemistry and Immunology, Morehouse School of MedicineAtlanta, GA, USA

**Keywords:** cancer, cell death, immune response, low-dose radiotherapy, radiation therapy, tumor microenvironment

## Abstract

Chemotherapy and radiation therapy (RT) are standard therapeutic modalities for patients with cancers, and could induce various tumor cell death modalities, releasing tumor-derived antigens as well as danger signals that could either be captured for triggering anti-tumor immune response. Historic studies examining tissue and cellular responses to RT have predominantly focused on damage caused to proliferating malignant cells leading to their death. However, there is increasing evidence that RT also leads to significant alterations in the tumor microenvironment, particularly with respect to effects on immune cells and infiltrating tumors. This review will focus on immunologic consequences of RT and discuss the therapeutic reprogramming of immune responses in tumors and how it regulates efficacy and durability to RT.

## INTRODUCTION

The immune system maintains a complex regulatory balance, maintaining immunological composure despite powerful immunological stimuli. There can be intense immune activity at mucosal surfaces and relative inactivity in closely neighboring sites. Multiple inflammatory mechanisms control the location of immune responsiveness and serve to direct therapeutic responses to the site of immunological insult. The host remains in a state of controlled immune activity, regulating the initiation and termination of immune responses to prevent widespread pathology is exploited by tumors to overcome the immunogenicity caused by their antigenicity and aggressive growth. Despite the presence of immune suppression, even multiply treated patients with significant tumor burden are capable of generating *de novo* tumor-specific immune responses (Laheru et al., 2008). Over the course of radiation therapy (RT), patients have been shown to develop tumor antigen-specific immune responses that were not detectable before treatment demonstrating that immune suppression in cancer patients and any immune suppression caused by RT is relative rather than absolute (Nesslinger et al., 2007).

Ionizing radiation is a powerful cytotoxic force that can be manipulated to specifically kill cancer cells at target sites. In addition to the direct effect of radiation, focal radiation can have distant bystander effects that influence tumor growth outside of the irradiated region (Ohba et al., 1998). The abscopal bystander effect would be an important phenomenon, whether it is intended to target pre-existing distant metastases or to residual disease that was not removed by the primary therapy. RT is not always used alone and clinical translation of radiation therapies that incorporate immunotherapy must take into account their interaction with surgery or the multitude of chemotherapies. Both chemotherapy and RT impact growing cancers through their ability to induce cell death by disrupting various parameters of cell biology necessary for survival (Haynes et al., 2008; Tesniere et al., 2008; Zitvogel et al., 2008). Leukocytes detect cell death through immune-based receptors for molecules released by dying cells (often termed “danger signals”), such as Toll-like receptor 4 (TLR4) and its ligands including the high-mobility group box 1 protein (HMGB1; Apetoh et al., 2007).

Effective anti-tumor therapy should induce sufficient tumor cell death in order to release tumor-associated antigens (TAAs) as well as danger signals attracting professional antigen-presenting cells (APCs) phagocytes to uptake and present tumor antigen for specific adaptive immunity. Proper cell death modality should be triggered in both tumor cells, tumor stem cell, and stromal cells. RT clearly influences multiple immune-based programs in tissues, some of which lead to durable tumor regression, whereas others propel tumor development. It seems reasonable to conclude that identifying pathways mediating activation of myeloid-based protumor immunity induced by RT, will encourage development of novel therapeutics that suppress those activities to effectively bolster RT responses. Moreover, blockade of these protumor immune-based pathways may also present the opportunity to combine RT with anti-tumor immune-therapeutics to yield effective and durable suppression of tumors, resulting in improved outcomes for patients with cancer. In the palliative setting, for patients who have rituximab and chemotherapy-resistant disease and bulky tumors, low-dose RT (LDRT; <1.0 Gy) is an active and non-toxic treatment modality that might alleviate symptoms for long periods. Conventional RT remains potentially toxic, particularly for patients whose disease is located in certain sites. As with LDRT, rituximab induces apoptosis which is suspected to contribute to the induction of a specific anti-lymphoma immune response in mice (Franki et al., 2008).

## IMMUNOGENICITY OF CHEMOTHERAPY AND RADIOTHERAPY

**FIGURE 1:**
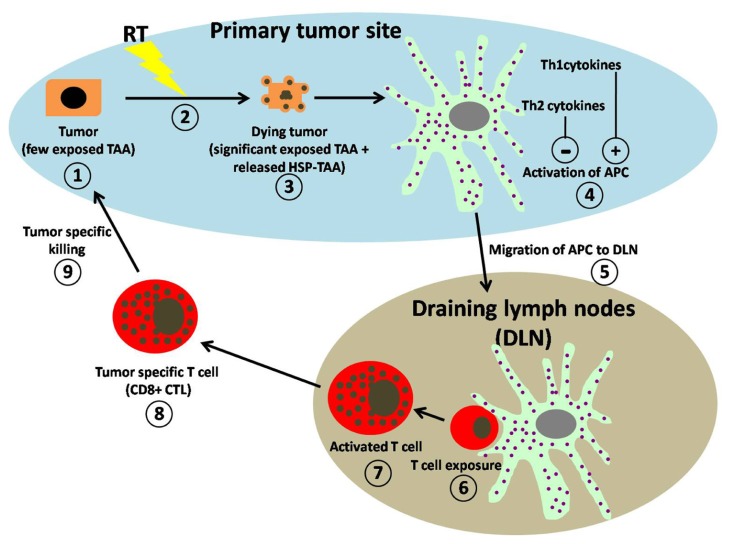
**Schematic representation of working hypothesis for RT-induced anti-tumor immune regulation.** (1) Within the primary tumor microenvironment (blue area) untreated tumors express few exposed tumor-associated antigens (TAAs). (2) Exposure to RT induces the (3) dying tumors to express significantly more TAAs on their surface and to release DAMPS, (4) which are both taken up by professional APCs resulting in their activation. Activation of APC is greatly enhanced (+) by the presence of Th1-type cytokines and significantly suppressed (−) by the presence of Th2-type cytokine. (5) Activated APCs migrate to draining lymph nodes (DLN; gray area). (6) Within the DLN, T cell exposure to APC is achieved by direct contact with activated APCs. (7) Activated T cells increase in size and granularity. (8) The activated T cells migrate from the DLN as tumor-specific T cells (CD8^+^ CTL) into the tumor microenvironment. (9) Within the tumor microenvironment CD8^+^ CTL perform tumor-specific killing.

Cancer research has primarily focused on the role of activating and/or inactivating mutations in genes regulating aspects of cell proliferation or cell death. Solid tumors contain neoplastic and non-neoplastic stromal cells embedded in a dynamic extracellular matrix (ECM) microenvironment. Cellular components of tumor stroma include hematogenous and lymphatic vascular cells, infiltrating and resident leukocytes, various populations of fibroblasts and mesenchymal support cells unique to each tissue microenvironment. Increased presence of extra follicular B cells, T regulatory cells (Tregs) and high ratios of CD4/CD8 and Th1/Th2 T lymphocytes in primary tumors or in draining lymph nodes correlates with tumor grade, stage, and overall survival (OS; Bates et al., 2006). Infiltration of macrophages into the tumor microenvironment particularly when CD8^+^ cytotoxic lymphocytes (CTL) are also present correlates with increased OS (Kawai et al., 2008). Macrophages exposed to Th1 cytokines including interferon-gamma (IFN-γ), tumor necrosis factor alpha (TNF-α), and granulocyte monocyte-colony stimulating factor (GM-CSF) exhibit enhanced cytotoxic activity, production of pro-inflammatory cytokines, and antigen presentation (**Figure 1**; Mantovani et al., 2007). On the other hand, macrophages exposed to Th2 cytokines such as interleukin-4 (IL-4) and IL-13, immune complexes or immunosuppressive cytokines instead block CTL activity and promote angiogenesis and tissue remodeling (**Figure 1**; Ruffell et al., 2010). The immunological clinical success story in metastatic melanoma is high-dose IL-2, which causes durable regression of significant disease in a subpopulation of patients (Rosenberg et al., 1998). Phase I trials with chemotherapy-induced lymphodepletion and adoptive transfer have been performed with impressive results, showing a 50% response rate in patients with stage IV cancer (Dudley et al., 2005). Preclinical data suggested an enhanced benefit associated with lymphoablative doses of radiation requiring hematopoietic stem cell rescue and these data have been confirmed in clinical studies where the addition of myeloablative radiation with hematopoietic stem cell rescue increased response rates to 72% (Muranski et al., 2006).

Radiation can replicate the effect of vaccination by providing an alternate means to present tumor antigens, it is important to examine why vaccination and RT synergize. Chakraborty et al. (2004) demonstrated that RT influenced the tumor site to render cancer cells more susceptible to T cell-mediated cytotoxicity, potentially through upregulation of a range of adhesion molecules (Garnett et al., 2004). Radiation has been shown to increase the expression of major histocompatibility complex (MHC) class I, and accentuating this effect *via* gene therapy increases the therapeutic margin of radiation. While radiation may have a direct effect on MHC expression, tumor antigen-specific cells elicited by radiation can upregulate IFN-γ in the tumor, and responsiveness to IFN-γ has been shown to be required for radiation-induced MHC upregulation. Combination of radiation-induced local inflammation and tumor-specific effector T cells can together alter the tumor vasculature, providing an additional mechanism of tumor control (Ganss et al., 2002). In prostate cancer patients, Gulley et al. (2005) demonstrated that vaccination with an immunogenic virus combination expressing the prostate serum antigen (PSA), in combination with radiation and IL-2, resulted in prostate-specific immune responses. Direct injection of dendritic cells (DCs) into tumors undergoing radiation has been shown to increase tumor-specific T cell priming and extend survival in murine models (Teitz-Tennenbaum et al., 2008). CD8 T cell responses play an important role in the therapeutic outcome of RT in immune-competent animal models suggesting that therapies targeting T cells have the potential to enhance this component of therapy in patients (Lee et al., 2009). Chemotherapy and RT are commonly believed to kill cancer cells by apoptosis, which is generally considered as non-immunogenic. Irradiated tumor cells (cell lines or autologous dissociated tumor pieces) engineered to secrete GM-CSF are able to mobilize DCs, plasma cells, invariant natural killer (NK) T cells, and tumor-reactive CD4^+^ and CD8^+^ T cells, both in mice and in metastatic cancer patients (Hodi and Dranoff, 2006). Many chemotherapeutic agents used to treat malignant diseases damage lymphocytes and consequently suppress cell-mediated immunity. New cancer treatment agents such as tyrosine kinase inhibitors, thalidomide and its derivatives, proteasome inhibitors, and IFNs have been found to have diverse immunomodulatory activities blocking immune surveillance of the malignancy and permitting disease recurrence, or, favorably, by reprogramming immunity to increase autologous anti-tumor effects.

Traditional fractionated radiation is locally immunosuppressive, dampening local immune responses as they develop due in part to the fact that lymphocytes are sensitive to radiation doses and are cleared rapidly from the radiation field (Rosen et al., 1999). Fractionation makes it possible to achieve a therapeutic dose of radiation to cancer cells while relatively sparing normal tissues from late toxicities. For example, in standard fractionation for breast cancer, the radiation dose may be given in 1.8 Gy doses daily for 6–7 weeks. A single radiation dose of 1.8 Gy will result in minimal toxicity to normal tissues in the region of the tumor, with one of the notable exceptions being lymphocytes. Treatment plans go to significant lengths to minimize dose to radiosensitive populations outside of the tumor. Tumor antigen-specific T cells can be isolated from many tumors, amplified *in vitro* and restored to full cytolytic function and tumor-draining lymph nodes are a rich source of tumor antigen-specific T cells (Robbins et al., 1996). Therefore, continued tumor radiation for 7 weeks would severely diminish tumor antigen-specific T cell populations through constant site-specific cytotoxicity. T lymphocytes are exquisitely sensitive to ionizing radiation with their high turnover and radiation sensitivity, are effectively ablated by relatively low radiation doses in an effective immunosuppressive therapy. Depletion of Tregs can remove this suppressive mechanism and restore anti-tumor immunity (Onizuka et al., 1999). Any T cells introduced into a T cell-deficient environment find themselves replete in both stimuli, resulting in homeostatic proliferative expansion to steady-state levels. In the process, T cells take on an activated phenotype, and have increased cytolytic activity to self and thus tumor antigens (Gattinoni et al., 2005). It was shown that in some mouse tumor models complete tumor regression was achieved following total-body irradiation (TBI). Gattinoni et al. (2005) likewise were able to show a significant increase in anti-tumor immunity associated with radiation-induced lymphodepletion and importantly, that radiation-induced lymphodepletion had a greater therapeutic benefit than was observed in a genetically deficient Rag1-lymphodepletion model. Immunostimulatory cytokines including IL-2, IL-12, and TNF-α have been used in combination with RT to stimulate anti-tumor T cell responses. Addition of these pro-inflammatory cytokines enhances RT efficacy by bolstering CTL cell responses (**Figure 1**). Interestingly, IL-3, a cytokine that activates monocytes and mast cells, delays tumor growth in response to RT (Oh et al., 2004). Intratumoral injection of CpG oligodeoxynucleotides that activate TLR9 on macrophages and DCs resulted in increased RT response and resistance to a second challenge with the same tumor, thus indicating development of a durable immune response. Antigen presentation on the surface of DCs to T cells requires both MHC and costimulatory molecules, B7 molecules and OX40. Inhibition of tumor growth and enhanced OS was also observed in a murine sarcoma model when RT was given in combination with an agonistic antibody for OX40, a costimulatory molecule found on activated T cells that stimulates T cell proliferation and differentiation. Inhibition of CTLA-4 co-stimulation also enhanced effectiveness of RT in 4T1 murine mammary carcinomas carcinoma resulting in diminished metastasis and increased survival, however, RT dose and timing were critical with regards to anti-CTLA-4 therapy (Dewan et al., 2009).

Cytokines are peptide-type regulatory proteins, such as the ILs and lymphokines, released by immune cells leading to generation of an immune response. Some cytokines act to inhibit immune responses, e.g., IL-10 and TGF-β, or instead stimulate immune responses, e.g., TNF-α or IL-1 (Germano et al., 2008). TNF-α mRNA and protein levels were increased in human sarcoma cells following RT, an effect that sensitized tumor cells to radiation-induced cell death. Macrophage-derived IL-1α and IL-1β have also been found increased in response to RT *in vivo* following sublethal TBI, as also have IL-6 and TGF-β. Consequences resulting from the release of these cytokines are recruitment and activation of leukocytes from peripheral blood and extravasation into tissue (tumor) parenchyma. Cell adhesion molecules such as ICAM-1, E-selectin and vascular cell adhesion molecule 1 (VCAM-1) are upregulated on endothelial cells during inflammation and are critical for leukocyte trafficking across endothelial barriers. Vascular endothelial cells within tumor vessels respond to RT by upregulation of ICAM-1 and E-selectin and thereby facilitate leukocyte arrest and adhesion prior to transmigration. Blockade of CD11b, the ligand for ICAM-1, in a transplantable murine squamous carcinoma model significantly reduced tumor-infiltration by CD11b^+^ myeloid cells following RT resulting in diminished tumor growth (Ahn et al., 2010). Similarly, examination of tumor tissue removed from head and neck cancer patients following RT revealed marked increase in endothelial ICAM-1 expression, in concert with increased β2 integrin-positive myeloid cell infiltration. The radiation-induced CXCL16 is an important mechanism by which RT promotes CD8^+^ T cell infiltration leading to tumor suppression. Stromal cell-derived factor-1 alpha (SDF-1α) is also upregulated following RT in bone marrow-derived cells and cell lines derived from brain tumors. Inhibition of the SDF-1α pathway with a small molecule inhibitor blocking the interaction of SDF-1α and CXCR4 prevented infiltration of macrophages and significantly delayed tumor regrowth following RT (Kozin et al., 2010). Vaccination of prostate cancer patients with recombinant viral-based vaccines expressing PSA, in combination with the costimulatory molecule B7-1 and standard RT to the prostate (70 Gy of RT in 1.8–2 Gy fractions), resulted in a threefold increase in PSA specific T cells and evidence of generating T cells against other prostate-specific antigens in 76% of patients (Gulley et al., 2005).

Stereotactic body RT (SBRT)-dose radiation has been shown to generate tumor antigen-specific T cells in mice bearing B16 tumors (Lugade et al., 2005), and comparable radiation doses are less effective at tumor therapy when conducted in immunodeficient mice (Lee et al., 2009). When using aggressive, transplantable tumor models where standard 1.8–2 Gy fractions are less effective, and tumor-bearing survival is too short to complete an extended fractionation schedule. A fundamental issue in stereotactic ablative radiotherapy (SABR) is whether classical radiobiologic modeling with the linear-quadratic (LQ) model is a valid method to assess the biologically effective dose at the high doses typically encountered in radiosurgery. The robustness of the LQ model to predict fractionation and dose-rate effects in experimental models *in vitro* and *in vivo* at doses up to 10 Gy is based on the premise that cell killing is the dominant process mediating the radio-therapeutic response for both early and late effects including vascular effects. However, the administration of a single high-dose of radiation *in vivo* had a much greater effect than predicted by the LQ model; they cited several examples including Leith et al. (1994) who calculated that the dose to obtain a high probability of tumor control for brain lesions would be at least 25–35 Gy using the LQ model, which was much higher than the observed clinically effective radio-surgical dose, which was in the range of 15–20 Gy. The *in vitro* survival curve has goodness of fit in all clinically significant ranges including the ablative range characteristic of SABR (Guerrero and Li, 2004).

## RADIATION THERAPY AND ACTIVATION OF STRESS-RESPONSE PATHWAYS

The delivery of an ablative dose of radiation of 15–25 Gy was found to cause a significant increase in T cell priming in draining lymphoid tissue, leading to reduction or eradication of the primary tumor or distant metastasis in a CD8^+^ T cell-dependent fashion in an animal model. While conventional 2 Gy doses seem inferior at generating such responses, higher sized dose fractions may be better than single doses (Dewan et al., 2009). Radiation cannot only kill tumor cells releasing tumor antigens and molecules with what are collectively called damage-associated molecular patterns (DAMPs) that exert various immunomodulatory effects including induction of the expression of cytokines, chemokines, and release of inflammatory mediators (**Figure 1**; Gattinoni et al., 2005). Radiation also increases the permeability of the local vasculature either directly or through cytokine production that leads to recruitment of circulating leukocytes into surrounding tissues including APCs and effector T cells (Ganss et al., 2002). Thus, a radiation-induced pro-inflammatory microenvironment within irradiated tumors could provide DCs with maturation inducing stimuli critical for eliciting effective antigen presentation (**Figure 1**). The introduction of cytokines, in particular IL-2 for cancer treatment was a major clinical effort that had modest success. This situation changed with the molecular cloning of human TAAs that could be recognized by T cells, the ability to culture powerful APCs in the form of DCs and to assess immune responses to specific tumor epitopes using tetramer and enzyme-linked immunosorbent spot (ELISPOT) assays (Yee et al., 2001). The “danger” model of immunity suggests that pathogens with associated molecular patterns (PAMPs) and DAMPS engender an inflammatory milieu that promotes the development of antigen-specific immunity through DC maturation that allows internalization of apoptotic and necrotic cellular debris and presentation of processed antigen to T cells. Thus, administration of radiation may therefore be considered to create an inflammatory setting via DC maturation, induction of apoptosis, necrosis, cell surface molecules, and secretory molecules (**Figure 1**). As with many other challenges, radiation upregulates expression of immunomodulatory surface molecules (MHC, costimulatory molecules, adhesion molecules, death receptors, heat shock proteins) and secretory molecules (cytokines, inflammatory mediators) in tumor, stromal, and vascular endothelial cells. Important amongst these may be the upregulation of TNF family members that could promote cell killing, not only by TNF in the microenvironment but also by radiation-induced TNF.

Activation of Fas-mediated cell death is a mechanism by which immune cells eliminate damaged cells, including those damaged by RT. Thus, while whole-body radiation is “immunosuppressive” due to triggering widespread apoptosis of immune cells via Fas, focal radiation such as that used for treatment of many types of solid tumors instead has limited immunosuppressive side effects, and may actually promote changes in the local tumor microenvironment that paradoxically enhance infiltration and activation of multiple immune cell types that may either foster, and/or suppress tumor development (de Visser et al., 2006). At the most simplistic level, a main mechanism by which ionizing radiation mediates a biologic effect is via generation of free radicals that lead to genotoxic (DNA) damage, and subsequent activation of stress-response pathways through activation of the DNA damage pathway ataxia telangiectasia mutated (ATM). Activation of the ATM protein pathway following RT involves activation of p53 and nuclear factor (NF)-κB transcription factors. NF-κB can also be activated independently of DNA damage through radiation-induced activation of TNFR-associated factors (TRAFs; Rashi-Elkeles et al., 2006). NF-κB directly regulates expression of molecules that promote a pro-inflammatory immune response, including TNF-α, IL-1 (Mori and Prager, 1996), chemokines such as CCL5 (Wickremasinghe et al., 2004); ICAM-1, E-selectin and VCAM-1, as well as MHC molecules, and expression of several anti-apoptotic genes including Bax and Bcl-2.

## ANTI-TUMOR THERAPY AND TUMOR MICROENVIRONMENT

The tumor microenvironment contains innate immune cells [NK cells, neutrophils, macrophages, mast cells, myeloid-derived suppressor cells (MDSCs), and DC] and adaptive immune cells (T and B lymphocytes) in addition to tumor cells and their surrounding stroma (fibroblasts, endothelial cells, pericytes, and mesenchymal cells; de Visser et al., 2006). Myeloma cells treated with low doses of common therapeutic agents, such as doxorubicin, melphalan, and bortezomib, upregulate DNAM-1 and NKG2D ligands. Azacytidine enhances tumor antigenicity by upregulating MHC class I and tumor antigen expression, increasing the release of pro-inflammatory cytokines and danger signals, and promoting antigen uptake by DC and killing by NK cells. Infiltration of the primary tumor by memory T cells, particularly of the Th1 and cytotoxic types, is the strongest prognostic factor in terms of disease-free and OS at all stages of clinical disease (Pages et al., 2010). A combined therapy of local radiation with Th1 cell could augment the generation of tumor-specific CTL at the tumor site and might also be effective for the treatment of distant metastases. The suppressive activity of MDSCs is associated with the intracellular metabolism of L-arginine, which serves as a substrate for inducible nitric oxide synthase (iNOS/NOS2) that generates NO and arginase 1 (ARG 1) which converts L-arginine into urea and L-ornithine. Tumor-derived exosome-associated Hsp72 could trigger Stat3 activation in MDSCs and determine their suppressive activity in a TLR2/MyD88-dependent manner (Chalmin et al., 2010).

## INTERACTION BETWEEN TUMOR CELLS AND THE IMMUNE SYSTEM

The tumor stroma plays an important role in the response to high-dose per fraction radiation treatment because the vascular endothelial cell apoptosis is rapidly activated above 10 Gy per fraction (Garcia-Barros et al., 2003), and that the ceramide pathway orchestrated by acid sphingomyelinase (ASMase) operates as a rheostat that regulates the balance between endothelial survival and death and thus tumor response (Truman et al., 2010). Damage to vascular/stromal elements in tumors has also been observed around 2 weeks after radiation exposure that was less dependent on size of dose per fraction (Chen et al., 2009). Pathological observations show profound changes in vasculature after radiosurgery and from studies on arteriovenous malformations, where obliteration of abnormal vasculature occurs months after irradiation, but is rarely seen below single doses of 12 Gy climbing steeply with increasing doses above this threshold (Szeifert et al., 2007). Although lymphocyte radiosensitivity is well recognized, the effects of different doses and delivery methods on systemic and locoregional naive, effector, or Treg or other immunologically relevant populations is still the subject of debate. Several authors have investigated the potential immunomodulatory effects of localized RT on tumors resulting in conflicting reports as to whether these responses promote or interfere with tumor reduction. This dualism is something that is to be expected and is inherent in a system that has to promote both destruction of pathogens and tissue healing while regulating anti-self-reactivity. Apetoh et al. (2007) showed that radiation can trigger signals that stimulate TLR4 on antigen-presenting DCs. Liao et al. (2009) have shown that irradiation of DC can enhance presentation of antigenic peptides by the exogenous pathway and is a maturation signal, while inhibiting internal antigen processing, and Merrick et al. (2005) have shown a decrease in IL-12 production that has a negative effect on presentation. Several reports have shown increased expression of MHC class I and coaccessory molecules after radiation of both tumor and host cells, while Chakraborty et al. (2004) reported a direct effect of radiation on tumors by modifying the phenotype of tumor cells to render them more susceptible to vaccine-mediated T cell killing, and others have shown that radiation-induced changes in the tumor immune microenvironment to promotes greater infiltration of immune effector cells (Lugade et al., 2005).

## CELL DEATH AND ANTIGEN RELEASE

Effective initiation of adaptive immune responses to cell-associated antigen requires presentation of antigen by professional APCs. Macrophages cross-present antigen from their environment and express an important selection of critical costimulatory molecules; however, for effective presentation of antigens to naive T cells in draining lymph nodes, DCs are particularly critical. For example, apoptotic cells efficiently load DCs with tumor antigen, but do not cause DC maturation (Melero et al., 2000). By contrast, antigen from non-apoptotic cells also loads DCs, but also causes DCs to mature and upregulate costimulatory molecules (Basu et al., 2000). These authors demonstrate that CD8 T cell responses play an important role in the therapeutic outcome of RT in animal models (Lee et al., 2009). That immune responses may already be relevant in the success of RT suggests we have an opportunity to increase the therapeutic margin of RT by further enhancing the immune component.

Low-dose RT induces the apoptosis of lymphoma cells. Primary lymphoma cell culture has shown an increase in the number of apoptotic cells after RT (Dubray et al., 1998). Another study using fine-needle cytology was coupled with *in vivo* imaging with 99Tc-Annexin-V scintigraphy in 11 follicular lymphoma patients, out of which, 10 patients were concordant with cytology in both the pre- and post-LDRT evaluation. These studies suggest that LDRT neutralizes the anti-apoptotic properties of the characteristic overexpression of Bcl-2 in follicular lymphoma cells (Langenau et al., 2005). The overexpression of p53 induced by LDRT was confirmed by p53-immunostaining with p53 expression, increasing from 5% of lymphoma cells to >80% after LDRT. This induction of the p53 target was seen as the dominant component of tumor cell apoptosis (Knoops and de Jong, 2008). The investigators showed that LDRT induced both intrinsic and prominently extrinsic apoptosis pathways (Knoops et al., 2007). Therefore, they found an upregulation of BBC3 (Puma), BAX, PMAIP1 (Noxa), and a significant overexpression of cleaved caspase-9 after LDRT. Death receptor genes, including TNFRSF10B (TRAIL-R2) and FAS, were also upregulated after LDRT. Knoops et al. (2007) also demonstrated that LDRT induced an upregulation of macrophage activation-related genes, indicating that macrophage activation was probably induced by signals from apoptotic cells. No increase of CD68^+^ cells was observed after LDRT. These data indicate that LDRT induces an apoptosis of follicular lymphoma cells that could activate innate and adaptive immunity and contribute to the therapeutic effect observed in clinical practice.

TLR4 expression by DCs also appears to be a prerequisite for efficient antigen presentation of tumor antigens furnished by dying cancer cells (Apetoh et al., 2007). Th1-related genes, including IL-18-, COP1-, and IFN-induced chemokines such as CXCL9, CXCL10, and CXCL11, were also induced after LDRT (Knoops et al., 2007). The response of RT outside of the radiation field, rarely observed in many malignancies including lymphoid malignancies due to the abscopal effect (Robin et al., 1981). The abscopal effect is not often observed after RT alone (Kusmartsev and Gabrilovich, 2002). Evidence supporting the role of RT in promoting cross-priming and the induction of anti-tumor T cell responses was suggested by at least one experimental model (Chakravarty et al., 1999). When Fms-like tyrosine kinase receptor 3 ligand (Flt3-L), a growth factor that stimulates the production of DCs were administered after the treatment of a mouse lung carcinoma by local RT, the treated mice experienced significant prolonged disease-free survival. In contrast, Flt3-L alone induced moderate delayed growth only. Mice bearing a syngeneic mammary carcinoma, 67NR, in both flanks were treated with Flt3-L after local irradiation with a single dose of 2–6 Gy to only one of the two tumors. The growth of the non-irradiated tumor was also impaired by the combination of RT and Flt3-L; moreover, growth of a non-irradiated A20 lymphoma in the same mice harboring an irradiated 67NR tumor was not affected. Demaria et al. (2004) also showed that no such effect was observed in athymic mice. With a murine colon adenocarcinoma model, Reits et al. (2006) showed that radiation enhances MHC class I expression and that anti-tumor immunotherapy with adoptive CTL cells is active only when preceded by RT (8–10 Gy) of the primary tumor. Similar observations were more recently described by Shiraishi et al. (2008) with ECI301 (a chemokine secreted by various leukocytes, including T lymphocytes and activated macrophages and recruiting certain cells such as monocytes and DCs) after local irradiation of 6 Gy. Marked infiltration of CD4^+^ and CD8^+^ cells was observed, not only in the irradiated site, but also at the non-irradiated site. In a recent animal model study, Lee et al. (2009) observed that local RT on grafted tumors generates CD8^+^ T cell immunity to lead to tumor reduction, reduces local relapse, and even eradicates metastasis in some settings. An increase of infiltrating T cells in the tumor microenvironment and the draining lymphoid tissues was seen 1–2 weeks after treatment with higher radiation dose (15–20 Gy in one to four fractions).

Additional approaches to induce a CTL-mediated tumor cell killing are based on the *in vivo* activation of DCs, which should take up tumor antigens and consecutively present tumor peptides to T cells to achieve co-stimulation. However, macrophages recognize and phagocytose dying tumor cells swiftly and silently and thereby remove tumor antigens (Gaipl et al., 2007), which are recruited by find-me signals such as lysophosphatidylcholine (LysoPC) secreted by RT-induced apoptotic cells. Moreover, the latter may even cause caspase 3-dependent tumor cell repopulation by generating potent growth-stimulating signals (Huang et al., 2011). In order to enable enhanced access of DCs to RT-induced apoptotic and necrotic tumor cells is to block their clearance by macrophages with the PS-binding protein Annexin A5 (AnxA5; Bondanza et al., 2004; Frey et al., 2009). The growth of syngeneic tumors is significantly retarded by a single injection of AnxA5 around the tumor. The combination of RT with AnxA5 resulted in the most effective inhibition of tumor growth (Frey et al., 2009). *In vivo* experiments with immune-competent mice bearing syngeneic tumors have proven that AnxA5 increases the immunogenicity of tumor cells. The injection of irradiated tumor cells pre-incubated with AnxA5 cured established tumors in about 50% of the animals, while the injection of irradiated tumor cells only resulted in <10% of tumor free mice (Bondanza et al., 2004). Targeting of PS on therapy-induced dying and on viable metastatic cells could therefore both lead to efficient anti-tumor immune responses by promoting uptake of the tumor cells by DCs, to mention here one of multiple possible modes of action resulting from the shielding of PS (Frey et al., 2012).

## RADIATION THERAPY-INDUCED IMMUNOGENIC CELL DEATH

Radiation of tumor cells generally produces two responses: proliferative arrest (which in the case of senescence is indefinite) or cell death, which occurs by several mechanisms including apoptosis, necrosis, autophagy, or mitotic catastrophe (Galluzzi et al., 2007). Autophagy is marked by sequestration of large parts of the cytoplasm in autophagic vacuoles typically before cells undergo apoptosis. Finally, mitotic catastrophe is described by prolonged mitotic arrest with associated micro- and/or multinucleation prior to undergoing death. Radiation-mediated cell death is generally thought to occur primarily through either apoptosis or mitotic catastrophe. Immunization with tumor cells treated with either chemotherapy or RT prevented regrowth of tumors in ~30% of mice as compared to mice immunized with untreated tumor cells. When cells were harvested from draining lymph nodes in immunized mice, and re-challenged *ex vivo*, only lymph node cells from mice immunized with tumor cells treated with RT produced IFN-γ in response to re-challenge. Protective immunization in this scenario was dependent on the presence of TLR4 on DCs and its ligand HMGB1, both released by tumor cells following RT (Apetoh et al., 2007). Two other factors, calreticulin and ATP, also significantly contribute to immunogenic cell death, in a manner similar to HMGB1, where cell death triggers rapid translocation of calreticulin to the surface of cells thereby promoting antigen presentation by dying cells and DCs (Perez et al., 2009). Cytotoxic therapies (chemotherapy and RT) induce rapid release of ATP from cells. ATP acts on the P2X(7) purinergic receptor expressed by DCs, leading to activation of the NOD-like receptor family, pyrin domain containing-3 protein (NLRP3)-dependent caspase-1 activation complex also known as the inflammasome. Inflammasome activation leads to release of pro-inflammatory cytokines such as IL-1β, which are important for priming T cells. When components of this pathway (NLRP3, caspase-1 or IL-1R) are absent, reduced T cell responses toward cells killed by chemotherapy or RT are observed (Ghiringhelli et al., 2009), thus indicating that release of ATP from dying cells is a critical aspect of immunogenic cell death and anti-tumor immunity. Innate leukocytes, including DCs, macrophages, NK cells, and mast cells, are referred to as “first responders” to inflammatory mediators, largely based on the fact that they are often pre-stationed in tissues. RT induces opposing responses in tumors with regards to DCs: directly irradiated DCs are less effective APCs, however, the tumor microenvironment generated by RT enhances APC capabilities of DCs. Tumor-infiltrating macrophages, derived from circulating monocytes, make up a substantial component of the leukocyte infiltrate in solid tumors (Mantovani and Sica, 2010). Using human macrophage-derived cell lines, Lambert and Paulnock (1987) observed that RT enhanced macrophage cytolytic activity. Other groups have reported that low-dose whole-body RT increased expression of TLR4/MD2 and CD14 expression on murine peritoneal macrophages, leading to increased secretion of anti-tumor cytokines including IL-12 and IL-18, thus indicating that RT increases anti-tumor potential of macrophages (Shan et al., 2007).

Mast cells are pre-stationed in many tissues where they act as important sentinel cells capable of mounting rapid responses to tissue damage. Heissig et al. (2005) reported that LDRT fostered mast cell-dependent vascular regeneration in a limb ischemia model where RT promoted vascular endothelial growth factor (VEGF) production by mast cells in a matrix metalloproteinase-9 (MMP-9)-dependent manner. RT, through MMP-9 upregulated by VEGF in stromal and endothelial cells, induced release of Kit-ligand (KitL) and promoted migration of mast cells from bone marrow to the ischemic site similar to RT effects in the thoracic cavity where mast cell density increased in bronchoalveolar lavage fluid (Heissig et al., 2005). RT and adaptive immunity in experimental rodent models of cancer development, e.g., brain, sarcoma, lung, and breast, RT alone or in combination with DC or immunostimulatory therapies enhanced generation of anti-tumor responses mediated by CTL cells (Matsumura et al., 2008). RT alone can also stimulate anti-tumor T cell-based immunity when given at high doses by increasing the number of activated CD8^+^ T cells. In 4T1 mammary tumors, recruitment of CTL cells is dependent on CXCR6, a receptor for CXCL16. RT in combination with anti-CTLA-4 mAB increases recruitment of CXCR6^+^ CD8^+^ T cells (Matsumura et al., 2008). In orthotopically transplanted sarcoma and carcinomas, presence of macrophages was inversely correlated with tumor regression following RT (Milas et al., 1987). In melanoma, local RT of implanted tumors increased the number of APCs in draining lymph nodes and increased the number of CD11b^+^ cells in tumors (Lugade et al., 2005). CD11b^+^ myeloid cells (a portion of which are macrophages) contribute growth factors such as VEGF and MMP-9 that supports angiogenic programs in growing tumors. Preventing influx of CD11b^+^ myeloid cells following RT results in enhanced RT effects likely due to their increased expression of T cell suppressive molecules iNOS and ARG 1 (Meng et al., 2010). Examination of tumor cells exposed to ionizing radiation *in vitro* indicates that RT induces expression of NKG2D ligands, an activating receptor for NK cells (Kim et al., 2006).

Robust acute inflammation could be triggered by sterile cell death which induces DAMP exposed on the plasma membrane or secreted extracellularly. These cell-derived DAMP, such as uric acid, DNA (specifically unmethylated CpG-rich regions), HMGB1, SAP130, S100 proteins, and Hsp could stimulate an IL-1- and inflammasome-dependent response (Osterloh et al., 2008). Other pro-inflammatory stress molecules released by dying cells include Hsp70, a stress-response protein with a role in binding defective proteins and presenting them on the surface of cells. When exposed to RT, pancreatic and colon carcinoma cell release Hsp70, thereby targeting them for lysis by NK cells (Gehrmann et al., 2005). That NKG2D ligands and Hsp 70 render cells more susceptible to NK cell-mediated cytolysis indicates that RT-stimulated NK activity may be an important component of RT-induced immune responsiveness. Apoptotic microparticles could transfer chemokine receptors and arachidonic acid between cells, activates complement, promote leukocyte rolling, and stimulate the release of pro-inflammatory mediators. LysoPC, but none of the LysoPC metabolites or other lysophospholipids, represents the essential apoptotic attraction signal able to trigger a chemotactic response through phagocyte receptor G2A (Peter et al., 2008). The prototypical DAMP-HMGB1 is released with sustained autophagy, late apoptosis, and necrosis. HMGB1 could act as chemotactic and/or activating factors for macrophages, neutrophils, and DC (Apetoh et al., 2007). Forced expression of CD39 (NTPDase-1, an ecto-apyrase responsible for the degradation of NTP) could abrogate the chemoattractant activity of apoptotic cells (Elliott et al., 2009). Myeloid leukemia, migrating hematopoietic progenitors, and also solid tumors were found to overexpress CD47, resulting in a reduced uptake by SIRPα-expressing macrophages.

## CONCLUSION

To design effective therapies for the future, it remains to be determined to what degree this endogenous response can be relied upon to clear tumors once they have successfully emerged from immune control. The radiation is cytotoxic to lymphocytes, and it is possible that prolonged fractionation limits the capacity of adaptive immunity to influence the outcome of RT. On the other end of the radiation dose scale, the advents of hypofractionated RT may permit more direct translation of the lessons learnt in animal models into clinical research and minimize the negative effects of fractionated radiation on tumor-site immune responses. With the continuing evolution of technology in RT it may become more feasible to optimize the cytotoxic component of radiation while simultaneously taking into account optimal immune activation. LDRT is now widely acknowledged to be very active against indolent lymphomas and is a useful tool in the management of this disease with virtually no toxicity. In clinical practice, the best methods to develop active combinations with RT and current treatments such as rituximab, immunotherapy, and chemotherapy remain to be studied. Combination immunotherapy and radiation approaches are being translated into the clinic where intratumoral DCs injection with coordinated irradiation and introduction of autologous, unmanipulated DCs have been the subject of anti-tumor therapy. At present, SABR represents an exciting, effective, yet empirically designed RT. In addition, SABR could be optimized for use with immunotherapeutic approaches so as to better generate tumor antigen-specific cellular immunity.

## Conflict of Interest Statement

The authors declare that the research was conducted in the absence of any commercial or financial relationships that could be construed as a potential conflict of interest.

## Abbreviations

APCs: antigen-presenting cells
ATM: ataxia telangiectasia mutated
CTL: cytotoxic lymphocytes
DAMP: damage-associated molecular patterns
DCs: dendritic cells
Flt3-L: Fms-like tyrosine kinase receptor 3 ligand
GM-CSF: granulocyte monocyte-colony stimulating factor
HMGB1: high-mobility group box 1 protein
HSP: heat shock proteins
ICAM: intracellular adhesion molecule
IFN-γ: interferon-gamma
IL: interleukin
LDRT: low-dose radiation therapy
LQ: linear-quadratic
LysoPC: lysophosphatidylcholine
MDSCs: myeloid-derived suppressor cells
MHC: major histocompatibility complex
NF: nuclear factor
NK: natural killer
OS: overall survival
PSA: prostate serum antigen
RT: radiation therapy
SABR: stereotactic ablative radiotherapy
SBRT: stereotactic body radiation therapy
TBI: total-body irradiation
TLR: Toll-like receptor
TNF: tumor necrosis factor
Tregs: T regulatory cells.
